# Indolepropionic acid and novel lipid metabolites are associated with a lower risk of type 2 diabetes in the Finnish Diabetes Prevention Study

**DOI:** 10.1038/srep46337

**Published:** 2017-04-11

**Authors:** Vanessa D. de Mello, Jussi Paananen, Jaana Lindström, Maria A. Lankinen, Lin Shi, Johanna Kuusisto, Jussi Pihlajamäki, Seppo Auriola, Marko Lehtonen, Olov Rolandsson, Ingvar A. Bergdahl, Elise Nordin, Pirjo Ilanne-Parikka, Sirkka Keinänen-Kiukaanniemi, Rikard Landberg, Johan G. Eriksson, Jaakko Tuomilehto, Kati Hanhineva, Matti Uusitupa

**Affiliations:** 1 Institute of Public Health and Clinical Nutrition, Department of Clinical Nutrition, University of Eastern Finland, Kuopio, Finland; 2 Institute of Biomedicine, University of Eastern Finland, Kuopio, Finland; 3 Department of Chronic Disease Prevention, National Institute for Health and Welfare, Helsinki, Finland; 4 Department of Food Science, Uppsala BioCenter, Swedish University of Agricultural Sciences, Uppsala, Sweden; 5 Department of Medicine, University of Eastern Finland and Kuopio University Hospital, Finland; 6 Clinical Nutrition and Obesity Center, Kuopio University Hospital, Finland; 7 School of Pharmacy, University of Eastern Finland, Kuopio, Finland; 8 LC-MS Metabolomics Center, Biocenter Kuopio, Kuopio, Finland; 9 Department of Public Health and Clinical Medicine, Family Medicine, Umeå University, Umeå, Sweden; 10 Department of Biobank Research, Umeå University, Umeå, Sweden; 11 The Diabetes Centre, Finnish Diabetes Association, Tampere, Finland; 12 Science Center, Tampere University Hospital, Tampere, Finland; 13 Institute of Health Sciences, University of Oulu, Oulu, Finland; 14 Unit of General Practice, Oulu University Hospital, Oulu, Finland; 15 Unit of Nutritional Epidemiology, Institute of Environmental Medicine, Karolinska Institute, Stockholm, Sweden; 16 Department of General Practice and Primary Health, University of Helsinki, Helsinki, Finland; 17 Folkhälsan Research Center, Helsinki, Finland; 18 Unit of General Practice, Helsinki University Central Hospital, Helsinki, Finland; 19 Vaasa Central Hospital, Vaasa, Finland; 20 Center for Vascular Prevention, Danube-University Krems, Austria; 21 Saudi Diabetes Research Group, King Abdulaziz University, Jeddah, Saudi Arabia; 22 Research Unit, Kuopio University Hospital, Kuopio, Finland

## Abstract

Wide-scale profiling technologies including metabolomics broaden the possibility of novel discoveries related to the pathogenesis of type 2 diabetes (T2D). By applying non-targeted metabolomics approach, we investigated here whether serum metabolite profile predicts T2D in a well-characterized study population with impaired glucose tolerance by examining two groups of individuals who took part in the Finnish Diabetes Prevention Study (DPS); those who either early developed T2D (n = 96) or did not convert to T2D within the 15-year follow-up (n = 104). Several novel metabolites were associated with lower likelihood of developing T2D, including indole and lipid related metabolites. Higher indolepropionic acid was associated with reduced likelihood of T2D in the DPS. Interestingly, in those who remained free of T2D, indolepropionic acid and various lipid species were associated with better insulin secretion and sensitivity, respectively. Furthermore, these metabolites were negatively correlated with low-grade inflammation. We replicated the association between indolepropionic acid and T2D risk in one Finnish and one Swedish population. We suggest that indolepropionic acid, a gut microbiota-produced metabolite, is a potential biomarker for the development of T2D that may mediate its protective effect by preservation of β-cell function. Novel lipid metabolites associated with T2D may exert their effects partly through enhancing insulin sensitivity.

Well-established lifestyle, metabolic and genetic factors are currently used for stratifying people at high risk of developing type 2 diabetes (T2D). However, the metabolic basis and early molecular events related to the onset of the disease are still poorly understood. Therefore, there is a need to utilize novel technologies to broaden this understanding, ultimately improving the potential for early prevention and reducing disease incidence.

Metabolomics enables the concomitant measurement of low–molecular weight metabolites such as nutrient intermediates, lipids, hormones and other signaling molecules, and may also provide new insights into the pathogenesis of T2D^1^,^2^,^3^,^4^. In particular, the non-targeted metabolite profiling, an approach that allows the hypothesis-free assessment of a wide spectrum of metabolites resulting from endogenous metabolism, dietary intake and gut microbial activity^5^, has the potential to broaden the possibility of novel discoveries related to the pathogenesis of T2D.

In the Finnish Diabetes Prevention Study (DPS) population^6^, which recruited participants with impaired glucose tolerance (IGT), the lower risk of developing T2D was associated with better insulin sensitivity (IS) and preserved β-cell capacity probably achieved by changing lifestyles^7^. Therefore, by applying a non-targeted metabolomics approach, our primary aim was to identify novel metabolites that may predict the risk of T2D. Moreover, we sought to investigate if these metabolites would modify two basic mechanisms of T2D, i.e. insulin secretion capacity or insulin sensitivity.

## Results

### Characteristics of the DPS participants

Participants who developed T2D (cases) did not differ in age and sex distribution from those who did not develop diabetes (non-cases) (Table 1). However, cases were more obese and had more disturbances in insulin and glucose metabolism than non-cases at metabolomics sampling (1-year follow-up) (Table 1).

### Identified biomarkers and diabetes likelihood

**Figure 1:**
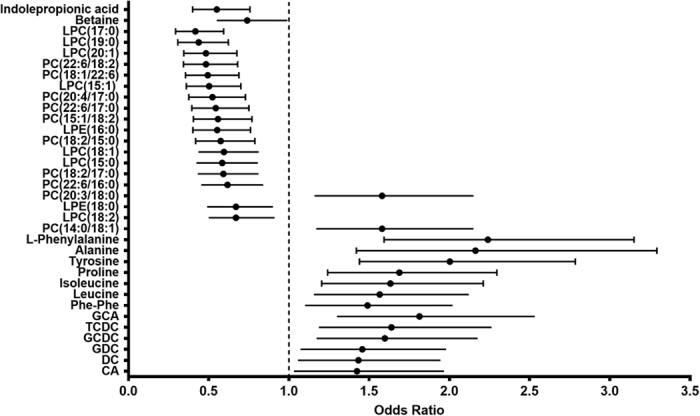
Identified metabolites and their association with the development of T2D in the DPS (N = 200). Closed bars: FDR-P < 0.05 Opened bars: P < 0.05. Phe: phenylalanine GCA: Glycocholic acid TCDC: Taurochenodeoxycholic acid GCDC: Glycochenodeoxycholic acid GDC: Glycodeoxycholic DC: Deoxycholic acid CA: Cholic acid.

Differential metabolic signatures were associated with a higher or lower likelihood of developing T2D (Fig. 1, Supplementary Table S1). The most prominent differences were found in several phosphatidylcholine (PC) lipid species and in indolepropionic acid, which were both inversely related to likelihood of developing T2D (Fig. 1). In contrast, certain amino acids and bile acids were increased in individuals who developed T2D during the first years of the DPS study (Fig. 1).

### Lipid-related metabolites protect from T2D

Six PCs and four lysoPCs (LPCs), and one lysophosphatidylethanolamine (LPE) were among the metabolites significantly inversely associated with diabetes at FDR-P < 0.05 (Fig. 1). Most of these lipids contained at least one of the following three fatty acids as part of the metabolite: C15:0 (pentadecanoic acid), C17:0 (heptadecanoic acid) or C15:1 (pentadecenoic acid). The very-long chain fatty acid C22:6 (docosahexaenoic acid) and the long chain fatty acid C18:2 (linoleic acid) were also frequently part of these protective lipids (Fig. 1).

### Amino acids and bile acid metabolites increase the likelihood of developing T2D

Several amino acids were significantly associated with T2D, for example phenylalanine and tyrosine (Fig. 1). The amino acids alanine, proline and isoleucine were also associated with increased risk of developing T2D (FDR-P < 0.05, Fig. 1).

Among the identified bile acids, the ones strongest associated with T2D are described in Fig. 1. They all nominally increased the likelihood of developing T2D (Fig. 1).

### Sensitive and post-hoc analyses

In sensitivity analyses, we excluded the participants who developed T2D during the first year of the follow-up. We observed similar chances of developing T2D according to each of the metabolites as in the whole study sample, even though the significances were attenuated (Supplementary Table S2).

In post-hoc analyses, adjustments for confounding factors such as BMI, fasting and post-load glucose and insulin at metabolomics sampling, or sex in the logistic regression models did not change the direction of the associations, even though, except for models including sex, the FDR-P values lost significance for most of the metabolites (Supplementary Tables S3–S5).

### Impact of lifestyle intervention on key metabolites

We also looked at the interaction between each metabolite and DPS study group (lifestyle or control) in the logistic regression models for the metabolites most strongly associated with T2D likelihood at FDR-P < 0.05. Interestingly, the group-wise stratification strengthened the protective role of the odd-chain lipids in the intervention group, while the branched-chain amino acids were linked with a higher chance of developing T2D only in the control group (Table 2). However, we only found an interaction between the study group for two lipids, PC(18:1/22:6) and LPC(19:0), whose inverse association with T2D risk was stronger in the intervention group, and between the study group with both tyrosine and proline, whose direct association with T2D risk remained significant only in the control group (Table 2). For any of the other metabolites, including indolepropionic acid, we did not find any interaction with the study group that could modify its association with the chance of developing T2D (Table 2).

### Indolepropionic acid associates with the course of insulin secretion during the follow-up in the DPS non-T2D cases

**Figure 2:**
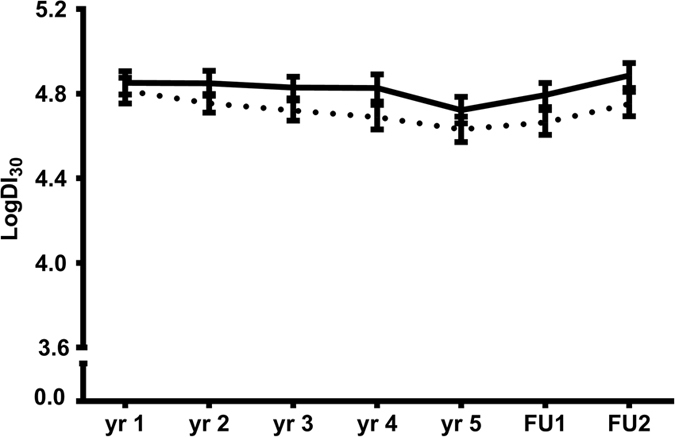
Association of indolepropionic acid with insulin secretion (DI_30_) in DPS. Descriptive figure of the course of DI_30_ during the follow-up according to the median cut-off point in indolepropionic acid in non-T2D cases. FU1: first post follow-up. FU2: second post follow-up. Solid line = above median cut-off; broken lines = below median cut-off. P = 0.04 for the difference between cut-off point groups.

After identifying the 17 strongest putative metabolites associated with T2D (Fig. 1), we examined whether these compounds may exert their effects by modulating IS (Matsuda ISI) or insulin secretion (DI_30_). Among non-cases, indolepropionic acid was directly associated with a better DI_30_ (β = 0.25 [0.06–0.44], P = 0.011) independently of the study group. However, indolepropionic acid was not associated with DI_30_ in study participants who developed T2D during the follow-up (Fig. 2).

Among study participants who remained non-diabetic (non-T2D cases), higher LPC(15:1), LPC(17:0), LPC(20:1), PC(22:6/18:2) and PC(15:1/18:2) were directly associated with better insulin sensitivity (Matsuda ISI) during the follow-up (β = 0.21 to 0.32, P = 0.039 to 0.001; Table 2), whereas higher isoleucine, phenylalanine and tyrosine were associated with lower Matsuda ISI (β = −0.23 to −0.36, P = 0.020 to 0.0003; Table 3). All above mentioned associations were independent of study group.

Among T2D cases, none of the metabolites were statistically significantly associated with DI_30_, but isoleucine, phenylalanine and tyrosine were inversely associated with Matsuda ISI during the follow-up (β = −0.26 to −0.40, P < 0.05; Table 3).

We next investigated whether insulin secretion, IS or their changes would modify the likelihood of developing T2D according to each of the metabolite mentioned above. Overall, the direction of the associations remained similar, but not all statistically significant, e.g. indolepropionic acid when adjusted for insulin secretion at follow-up (P = 0.09) and the amino acids tyrosine and isoleucine when adjusted either for IS (P = 0.27 and P = 0.40, respectively) or insulin secretion (P = 0.72 and P = 0.83, respectively).

### Indolepropionic acid and lipid metabolites are associated with high sensitive C-reactive protein (hsCRP) levels

Due to the interrelation among gut microbiota, low-grade inflammation and T2D, we examined if circulating levels of hsCRP would be related with indolepropionic acid or the lipid metabolites associated with T2D risk and either DI_30_ or Matsuda ISI during the follow-up.

Serum hsCRP was negatively correlated with indolepropionic acid at the time of sampling (r = −0.23, P = 0.006), independently of BMI (P = 0.03), both fasting (P = 0.009) and 2-h glucose (P = 0.02) and study group (P = 0.006). Nevertheless, adjustment for hsCRP in the statistical analyses did not modify the association of indolepropionic acid with insulin secretion in non-T2D cases (*β* = 0.31 [0.09–0.51], P = 0.004).

Similarly, hsCRP was negatively correlated with LPC(17:0): r = −0.42, P = 2 × 10^−7^; PC(22:6/18:2): r = −0.29, P = 3 × 10^−4^; LPC(15:1): r = −0.37, P = 5 × 10^−6^; LPC(20:1): r = −0.43, P = 5 × 10^−6^ and PC(15:1/18:2): r = −0.42, P = 2 × 10^−7^, mostly independent of BMI, fasting and 2-h glucose and study group (Supplementary Table S6). Even though hsCRP did not significantly modify the association of either LPC(15:1) or PC(15:1/18:2) with insulin sensitivity in non-T2D cases (β = 0.24 [0.04–0.45], P = 0.02 and β = 0.27 [0.02–0.52], P = 0.035, respectively), the effect of LPC(17:0), PC(22:6/18:2) and LPC(20:1) on Matsuda ISI was blunted by the inclusion of hsCRP in the models (P > 0.05 for all).

### Indolepropionic acid is associated with fiber intake

**Figure 3:**
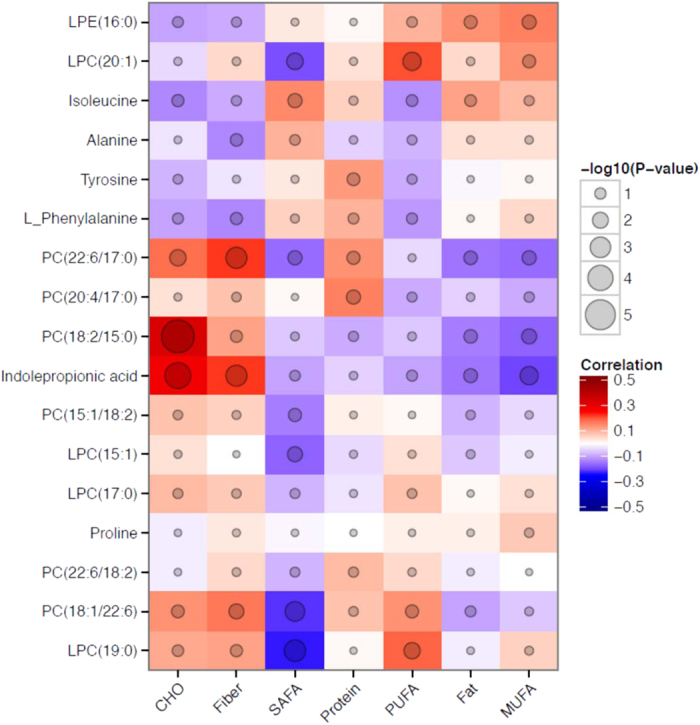
Correlation matrix (Pearson correlation coefficients) of identified top ranking metabolites with energy-adjusted dietary intake. CHO: carbohydrates; SAFA: saturated fat; MUFA: monounsaturated fat; PUFA: polyunsaturated fat.

We next examined the association between the identified metabolites and the intake of relevant nutrients cross-sectionally at the time of serum sampling. Indolepropionic acid was the metabolite most significantly and consistently related to both total carbohydrate and fiber intakes (r = 0.28, P = 9.1 × 10^−5^ and r = 0.23, P = 0.001; respectively). Overall, the majority of the lipids were negatively correlated with the intake of saturated fatty acids (Fig. 3 and Supplementary Table S7).

Additionally, we found that serum total alkylresorcinols and C17:0/C21:0 ratio, biomarkers of whole grain wheat and rye intake and the proportion of whole grain rye intake, respectively, were correlated with indolepropionic acid (r = 0.22, P = 0.003 and r = 0.23, P = 0.001; respectively), total fiber (r = 0.21, P = 0.003 and r = 0.28, P = 0.00006) and rye bread (r = 0.25, P = 0.0004 and r = 0.29, P = 0.00003) intakes.

### Indolepropionic acid and T2D in two other independent population-based studies

In order to test if the observed protective association of indolepropionic acid with the development of T2D could be repeated, we examined it in two population-based studies, METSIM^8^(Metabolic Syndrome in Men), and VIP (Västerbotten Intervention Program)^9^.

**Figure 4:**
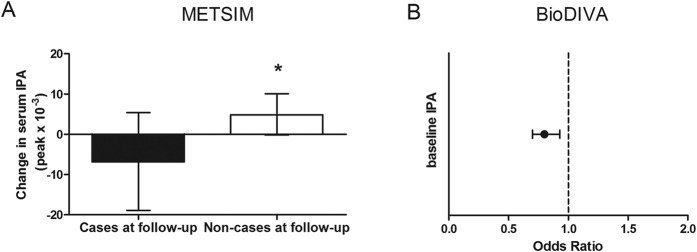
(**A**) Changes in indolepropionic acid (IPA) in cases and non-cases of T2D in METSIM study (n = 110). Changes are calculated as measurement at follow-up minus baseline and given as mean and 95% CI. The asterisk denotes P = 0.010 for the association between the changes in IPA and T2D (case or non-case at 5-year follow-up) after applying ANCOVA adjusted for baseline IPA. (**B**) Association of baseline IPA with T2D in BioDIVA study (P = 0.003, after applying conditional logistic regression, n = 1006).

METSIM is a prospective population-based study in Finnish men. We analysed samples from baseline and follow-up of 110 randomly selected participants free of T2D at baseline from which 55 were diagnosed with T2D at the 5-year follow-up. At baseline, there was no cross-sectional association between indolepropionic acid and T2D risk (P = 0.72), but during the 5-year follow-up indolepropionic acid was lower in subjects who developed T2D than in those who remained non-diabetic (P = 0.027) and they had decreased level of indolepropionic acid during the follow-up whereas in non-cases it was increased (Fig. 4A). Moreover, an increase in indolepropionic acid during the 5-year follow-up was inversely associated with the likelihood of developing T2D (OR: 0.31 [0.12–0.76], P = 0.01). Both associations, however, did not remain after controlling for BMI at baseline or at follow-up (P = 0.06 for the association of indolepropionic at follow-up with T2D in both models) or after controlling for BMI changes (P = 0.20 for the association of changes in indolepropionic and T2D).

After finding a suggestive, yet significant inverse association between the change in indolepropionic acid level and the likelihood of developing T2D in Finnish men, we wanted to further analyse it in a larger independent Swedish population including both genders. VIP is a Swedish population-based prospective cohort, within which the diabetes registry DiabNorth has identified individuals with diabetes. A case-control study, BioDIVA (Biomarker Discovery and Validation) within this cohort, comprises 503 incident T2D cases and their individually matched healthy controls. In BioDIVA indolepropionic acid was about 15% lower in T2D cases than in their matched healthy controls at baseline (P = 0.0032) and it was negatively associated with T2D incidence (OR: 0.80 [0.70, 0.93], P = 0.003, Fig. 4B) in a crude model, and more importantly even after adjustment for BMI (OR: 0.74 [0.62.0.89], P = 0.001) and further adjustment for fasting glucose (OR: 0.73 [0.61.0.88], P = 0.001). Moreover, ORs were re-calculated after excluding 95 pairs where participants had abnormal fasting glucose (≥5.9 mmol/L) or 2-h glucose at OGTT (2-h > 11.1 mmol/L) at baseline, or where cases developed diabetes during the first two years after baseline sampling. This did not substantially affect the associations between indolepropionic acid and T2D risk. Furthermore, similarly as in DPS, indolepropionic acid was positively correlated with dietary fibre intake at baseline (r = 0.16, P = 5.4 × 10^−7^).

## Discussion

**Figure 5:**
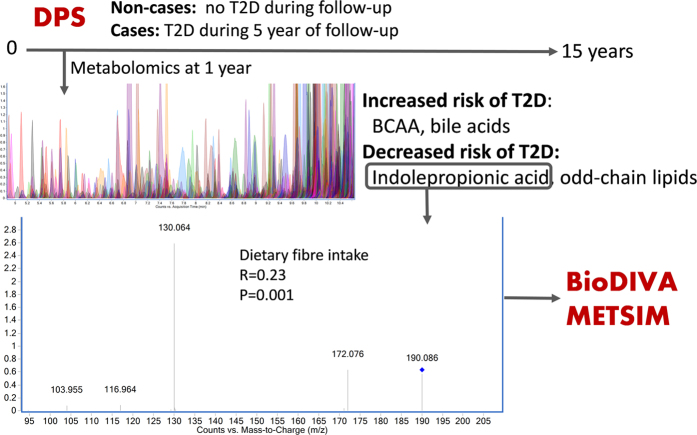
Summary of the study set-up and major findings. The non-targeted LC-MS based metabolite profiling was conducted within the Finnish Diabetes Prevention Study (DPS) by examining two groups of individuals who took part in the (DPS); those who either early (within five years) developed T2D (n = 96) or did not convert to T2D within the 15-year follow-up (n = 104). Key findings included the inverse association of indolepropionic acid with T2D risk. This finding was replicated in two additional cohorts, Biomarker Discovery and Validation (BioDIVA, 503 incident T2D cases and matched healthy controls), and Metabolic Syndrome in Men (METSIM, baseline and follow-up samples from 110 participants free of T2D at baseline from which 55 were diagnosed with T2D at the 5-year follow-up). Indolepropionic acid was associated with dietary fiber intake in the DPS and BioDIVA studies, whereas in METSIM such data was not available.

As summarized in Fig. 5, our findings show that indolepropionic acid, a gut microbial metabolite^10^,^11^,^12^, is associated with a reduced likelihood of progression to T2D in overweight individuals with IGT. In persons who did not develop diabetes within a 15-year follow-up, serum indolepropionic acid was associated with better preservation of β-cell function during the initial 7-year follow-up. Additionally, indolepropionic acid was directly associated with dietary fiber intake, suggesting a link between diet, intestinal microbiota, insulin and glucose metabolism and T2D risk. The suggestive protective role of indolepropionic acid was found also in another Finnish study, Metsim. Furthermore, these observations were replicated in a Swedish healthy population, as the baseline indolepropionic acid levels were associated with lower likelihood of future T2D, and likewise were correlated with dietary fiber intake. Interestingly, several novel PC species were also inversely associated to the development of T2D. Most of these lipids were associated with better insulin sensitivity in persons who did not develop T2D. Moreover, several amino acids and bile acids were associated with early development of T2D in line with previous studies^1^,^2^,^13^,^14^.

The putative protective effect of serum indolepropionic acid on the development of T2D may be explained firstly by its role in modulating incretin secretion from enteroendocrine L cells, more specifically, glucagon-like peptide (GLP)-1^15^. Incretin hormones, especially GLP-1, may play a critical role in the pathogenesis of T2D^16^. Secondly, indolepropionic acid has been shown to exert potent anti-oxidative stress capacity^17^,^18^, suggesting a possible role of this metabolite on protecting β-cell from damage associated with metabolic and oxidative stress, and possibly from amyloid accumulation^19^,^20^.

Diet is a major factor influencing the composition and metabolism of the colonic microbiota, which can elicit a wide range of systemic effects^21^,^22^,^23^. In our study, higher serum indolepropionic acid was directly associated with the intake of total fiber, mainly originated from whole grains. We confirmed this observation by serum alkylresorcinol measurements^24^. Indolepropionic acid is a microbiota-produced deamination product of the amino acid tryptophan. The type of carbohydrate ingested and pH level can affect the production of metabolites as indole compounds by the intestinal microflora^10^,^11^. We hypothesize that high intake of dietary fiber and whole grain cereal products may change gut microbiota towards a higher production of indolepropionic acid, thereby promoting preservation of insulin secretion capacity. This hypothesis fits well with previous observational findings on the protective role of fiber^25^ and low-fat high-complex carbohydrate diet for T2D^21^. The role of microbiota in *e.g*. efficient conversion of complex indigestible dietary carbohydrates into short-chain fatty acids and maintenance of gut microbiome carbohydrate fermentation seem to be important to maintain gut and systemic health^22^,^26^.

We identified several LPCs and PCs that were inversely associated with T2D incidence. The PCs are the major glycerophospholipids in eukaryotic cells and an essential component of cellular membranes. In animal models, LPCs have been reported to induce insulin secretion from pancreatic β-cells^27^, to directly activate glucose uptake by adipocytes and to lower blood glucose levels in models of Type 1 diabetes and T2D^28^. About half of the PCs identified in our study were directly associated with IS during the follow-up and inversely correlated with total saturated fat intake and circulating levels of hsCRP. Therefore, the protective effect of these metabolites on T2D might occur at least partly through its influence on IS^29^ and perhaps low-grade inflammation^30^,^31^.

The protective lipid metabolites in our study had mainly long-chain unsaturated and odd-chain fatty acids in their structure, e.g. 15:0 and 17:0. Odd-chain fatty acids in blood phospholipids are considered as biomarkers of dairy intake, although inconsistently, and have been related with reduced T2D incidence^32^,^33^,^34^. Gut microbiota is also related to the regulation of lipid homeostasis^35^, e.g. some lipid species, such as triacylglycerol containing odd-chain fatty acids, are linked to certain gut microbiota, and not necessarily to dietary fat intake^34^. In this regard, we found that indolepropionic acid was positively correlated with odd-chain fatty acid containing PCs, also suggesting that these metabolites could result from the metabolism of microbiota. Until now, only LPC18:2 and LPC17:0 have been reported in the literature to predict T2D^2^,^36^,^37^,^38^, and therefore our findings regarding the other lipid species are novel.

We also observed that certain serum amino acid metabolites already shown to be directly associated with T2D, insulin resistance and glucose metabolism^1^,^2^,^3^,^39^,^40^,^41^, increased the likelihood of T2D. Similarly, several metabolites identified as bile acids were related to the development of T2D and, overall, were positively correlated with the amino acids associated with T2D, especially with the ones putatively affecting IS. Interestingly, when addressing the likelihood of T2D separately in the control and lifestyle groups of the DPS, we found that in particular tyrosine and proline interacted with lifestyle, indicating that the deleterious metabolism resulting in their increase and consequent development of T2D can be diminished by lifestyle intervention that includes changes in body weight, exercise and the quality of diet.

Recently, the concept that bile acids can act as metabolic modulators of lipid and glucose metabolism has arisen^42^,^43^,^44^. Even though most of these bile acids lost their association with T2D after taking into account the effect of insulin and glucose metabolism, recent findings showed considerably increased values of most of these bile acids in plasma of T2D patients compared to healthy subjects^13^. In addition, the observed differences in the circulating bile acid levels in individuals at risk of T2D are likely attributed to the altered composition of gut microbiota^45^. Moreover, most of the top significant metabolites containing odd-chain fatty acids in our study were inversely associated with the bile acid metabolites related to increased T2D risk, therefore reinforcing that these lipid species may result from microbiota activity and not necessarily directly from dietary intake. Taken together, our results suggest that T2D is predicted by circulating metabolites reflecting gut microbiota composition and function. The observed inverse relationship of low-grade inflammation estimated by hsCRP with protective lipid metabolites and indolepropioinic acid support this view^46^,^47^.

Our study has some limitations. First of all, we did not have baseline samples available from the DPS study, and therefore we had to use the samples collected one year after the onset of the study. Additionally, insulin secretion and IS were not measured either by the hyperinsulinemic-euglycemic clamp or the intravenous glucose tolerance test (IVGTT). Instead, we used an IVGTT for validation of the indices^7^. Strengths of the present study include the well characterized and homogenous study population (obese, middle-aged individuals with IGT), and yearly measurements of insulin secretion and sensitivity estimates during a long period of follow-up of a carefully conducted lifestyle intervention study population. A particular strength of our study is that we were able to find a suggestive association between indolepropionic acid and the incidence of T2D among Finnish men in a small sub-sample of the Metsim study and finally to replicate the inverse association of indolepropionic acid with T2D risk in an independent study in a Swedish men and women. Furthermore, in that study indolepropionic acid was also associated with fiber intake. These results are convincingly suggesting a potential biological role for indolepropionic acid, and they highlight the importance of the explorative metabolite profiling approach in bringing out novel findings that can subsequently be addressed in focused examinations for replication and eventually validation.

In this study, we observed a link between diet - especially fiber, intestinal microbiota, insulin and glucose metabolism and T2D risk. We therefore propose that gut-microbiota derived indolepropionic acid is a compound that has a protective role concerning the development of T2D. The possible role of indolepropionic acid in mediating the association of preservation of β-cell function with lower risk of developing T2D, and of the specific lipid metabolites exerting their protective effects partly through enhancing insulin sensitivity and lowering inflammation require further investigation.

## Methods

### Study participants

The DPS was a randomized, controlled, multicenter study carried out in Finland between the years 1993 and 2001 (ClinicalTrials.gov NCT00518167). A total of 522 individuals with BMI > 25 kg/m^2^, age 40–64 years, and IGT based on the mean values of two 75 g glucose oral glucose tolerance tests (OGTT) and on WHO 1985 criteria were randomly allocated into either a lifestyle intervention or control group in five centers during 1993 to 1998 (Supplementary Fig. S1). After a mean four-year intervention (active study) period, the post-intervention follow-up was carried out with annual examinations. The DPS study design and methods have been reported in detail elsewhere^6^,^48^ and are briefly described in the online supplementary methods. The study protocol was approved by the Ethics Committee of the National Public Health Institute of Helsinki, Finland. The study design and procedures of the study were carried out in accordance with the principles of the Declaration of Helsinki. All study participants provided written informed consent.

In the DPS the main end-point was diagnosis of T2D defined by the WHO 1985 criteria (plasma fasting glucose ≥7.8 or 2-h glucose ≥11.1) to be confirmed by a repeated positive OGTT and verified by a physician. At baseline and at annual visits, individuals completed a medical history questionnaire and a 3-d dietary record, and underwent physical examination including anthropometric measurements and an OGTT^6^,^48^. The completeness of the food records was checked by the study nutritionist during each study visit^6^. In the present study, we also measured total alkylresorcinols and C17:0/C21:0 ratio, biomarkers of whole grain intake or relative whole grain rye intake, respectively, according to Wierzbicka *et al*.^49^, in serum samples at 1-year follow-up.

After the intervention (active study) period, the post-intervention follow-up was carried out with annual examinations. The individuals free of T2D participated in the post-intervention follow-up study at least once^6^.

### Study design

The present study was designed to include a selected subgroup of 200 participants from DPS (Table 1) who had fasting serum samples available from the first year visit of the active study period (1-year follow-up). These participants were either diagnosed with T2D during the first five years of the follow-up (N = 96; “cases”) or remained free of T2D (N = 104; “non-cases”) during the 15-year follow-up since the beginning of the DPS (Supplementary Fig. S1). The purpose of this exploratory design was to separate the extremes in terms of development of T2D within the follow-up to best characterize the early metabolic differences related to the increased risk of the disease.

### Laboratory determinations

Glucose and insulin levels were determined as previously described^7^. In the DPS, during 1993 to 1996 a baseline 2h-OGTT was performed (75 g oral glucose load) with fasting and 2-h samples for glucose and insulin and during follow-up visits starting from the middle of 1996, samples were also taken for 30 min insulin and glucose measurements^7^. High sensitive C-reactive protein was measured in fasting serum at metabolomics sampling using an IMMULITE^®^ 2000 Systems Analyzer according to the manufacturer instructions (Siemens Healthcare Diagnostics, Inc. Tarrytown, NY).

### Calculations

As surrogate indices of the first/early-phase insulin secretion and of peripheral IS we used the disposition index_30_ (DI_30_) and the Matsuda index of IS (Matsuda ISI), respectively, which were calculated as previously validated from an OGTT^7^,^50^,^51^.

In converters to T2D (cases) Matsuda ISI or DI_30_ annual values were averaged from the available yearly measurements at years 2, 3, 4 and 5, and in non-converters (non-cases), the first two post study follow-up measurements were also included.

### Non-targeted LC-MS metabolite profiling analysis

An aliquot (100 μL) of stored (−80 °C) fasting serum samples was mixed with 400 μL of acetonitrile (ACN; VWR International, Leuven, Belgium), and mixed in vortex at maximum speed 15 s, incubated on ice bath for 15 min to precipitate the proteins, and centrifuged at 16 000 × g for 10 min to collect the supernatant. The supernatant was filtered through 0.2 μm PTFE filters in a 96 well plate format. Aliquots of 2 μL were taken from at least half of the plasma samples, mixed together in one tube, and used as the quality control sample in the analysis. Additionally a solvent blank was prepared in the same manner.

The samples were analyzed by the UHPLC-qTOF-MS system (Agilent Technologies, Waldbronn, Karlsruhe, Germany) that consisted of a 1290 LC system, a Jetstream electrospray ionization (ESI) source, and a 6540 UHD accurate-mass qTOF spectrometerry. The samples were analyzed using two different chromatographic techniques, i.e. reversed phase (RP) and hydrophilic interaction (hilic) chromatography. Data were acquired in both positive (+) and negative (−) polarity. The sample tray was kept at 4 °C during the analysis. The data acquisition software was MassHunter Acquisition B.04.00 (Agilent Technologies). The quality control and the blank samples were injected after every 12 samples and also in the beginning of the analysis. The sample order of the analysis of the samples was randomized. Details on the technical procedures and parameters are described in the online supplementary methods.

### Data collection

Data were collected with “Find by Molecular Feature” algorithm in MassHunter Qualitative Analysis B.05.00 software (Agilent Technologies, USA). The extraction algorithm was set to collect peaks with threshold at 200 counts, and the allowed ion species were limited to [M + H]+ and [M + Na]+ in ESI(+), and [M−H]− and [M+Cl]−, in ESI(−). Only signals over compound height threshold of 2500 counts containing least with two ions were included in the compound list. Peak spacing tolerance for isotope grouping was 0.0025 m/z plus 7 ppm, with isotope model for common organic molecules. Data files (.cef-format) were exported to Mass Profiler Professional (Agilent Technologies) for peak alignment. After the first initial alignment, the data were combined in one.cef file, against which the original raw data was reanalyzed. For this recursive analysis, compound mass tolerance was±15 ppm, retention time±0.2 min and symmetric expansion value for chromatograms ±10 ppm. Resulting compounds were re-exported to Mass Profiler Professional software for peak alignment and data cleanup. The number of collected metabolite features from RP(+), RP(−), hilic(+), and hilic(−) was 2775, 2905, 1871, 1056, respectively.

In the case of the BioDIVA cohort (See “Validation cohorts”), the data collection and deconvolution was performed with XCMS^52^. The R program based pipeline, ‘batchCorr’ was used for alignment correction and within- and between-batch signal normalization^53^.

### Selection of metabolites to be identified

To account for non-normal distributions, metabolomics data were transformed using rank-based inverse normal transformation. Logistic regression for comparisons between T2D cases and non-cases as the dependent variable and further adjusted for study group was applied. The P-values were adjusted for multiple testing using Benjamini-Hochberg false discovery rate (FDR) within each analytical approach. FDR-P < 0.05 was considered to be statistically significant and a P < 0.05, nominally significant.

We first ranked the metabolites, according to their statistical significance applying the cut-off FDR-P < 0.05, as explained in the methods above. Additionally, the relative difference in average peak area value between cases and non-cases had to be at least 5%. In order to further remove noise and insignificant signals, only metabolic features found in at least 30 cases or controls with relative peak area abundance of >50000 counts were considered relevant. After this filtering procedure, the number of statistically significant metabolite features was 243, 176, 125, and 56, in RP(+), RP(−), hilic(+), and hilic(−), respectively. Notably, in the hilic(+) mode, altogether 53 signals were related to the hexose sugar, reflecting the disturbance of glucose metabolism related to T2D risk, and were not considered for further study. The differential metabolites were identified based on the MS/MS spectral comparison with pure standard compounds, or via search of the candidate compounds in the databases including the Human Metabolome database, METLIN, ChemSpider and SciFinder, and the results verified with the MS/MS spectral features included in the databases or reported in earlier publications. In Supplementary Table 1 we present the metabolites associated with T2D that survived correction for multiple comparisons and their corresponding identification level.

For the metabolites that remained significant after the adjustments for multiple testing, ANCOVA models were applied to examine the effect of each metabolite on the averaged subsequent study follow-up years Matsuda ISI or DI_30_ by T2D conversion group. Cases at 1-year follow-up were excluded for this purpose.

We also investigated the associations between metabolites and T2D by logistic regression models where we adjusted for the characteristics that differed significantly between cases and non-cases at serum sampling in order to control for confounding. Conditional logistic regression was used for testing the association between indolepropionic acid and T2D in BioDIVA study.

Cross-sectional correlations were calculated using Pearson’s product (r) between metabolites and dietary intake or hsCRP. For the correlation analyses, the mean daily nutrient intakes were previously energy-adjusted^54^ due to the high correlation between the intake of energy and each individual nutrients. A two-sided P value of <0.05 was considered statistically significant for all described secondary analyses.

### Validation cohorts

In order to test if the results obtained in relation to indolepropionic acid could be replicated, two dietary studies were examined.

A random selection of 110 subjects participating in the prospective population-based METSIM cohort, which includes 10,197 Finnish men aged 45–73 years and examined in 2005–2010 (See Supplementary Methods online)^8^ were analysed for the relationship between the metabolite indolepropionic acid and T2D development found in DPS. From these 110 participants who were free of T2D at baseline, 55 developed T2D (cases) and 55 remained free of T2D (control) during a mean of 5.9-year follow-up. Indolepropionic acid data derived from the non-targeted metabolomics analyses were available from baseline and follow-up. A written informed consent was obtained from all study subjects. The study was approved by the Ethics Committee of the University of Eastern Finland and Kuopio University Hospital. The study design and procedures of the study were carried out in accordance with the principles of the Declaration of Helsinki.

Additionally, a total number of 503 matched case-control pairs were included in the study BioDIVA, utilizing the DiabNorth diabetes registry to form a study nested within the Västerbotten Intervention Programme (VIP) cohort, which is one of the sub-cohorts of the Northern Sweden Health and Disease Study (NSHDS)^9^. Cases had a median follow-up time of 7-year before T2D diagnosis and were individually matched to healthy controls at baseline. A written informed consent was obtained from all study subjects. The study was approved by the regional ethical review board in Uppsala. The study design and procedures of the study were carried out in accordance with the principles of the Declaration of Helsinki.

## Additional Information

**How to cite this article**: de Mello, V. D. *et al*. Indolepropionic acid and novel lipid metabolites are associated with a lower risk of type 2 diabetes in the Finnish Diabetes Prevention Study. *Sci. Rep.* **7**, 46337; doi: 10.1038/srep46337 (2017).

**Publisher's note:** Springer Nature remains neutral with regard to jurisdictional claims in published maps and institutional affiliations.

## Supplementary Material

Supplementary Information

## Figures and Tables

**Table 1:** Characteristics of the participants at metabolomics sampling.

	Cases (N = 96)	Non-cases (N = 104)	P^*^
Study group (N, lifestyle/control)	37/59	62/42	0.003
Age (years)	55.3 ± 7.2	56.3 ± 6.6	0.29
Sex (male/female)	35/61	37/67	0.90
Body weight (kg)	90.0 ± 16.9	80.2 ± 12.1	<0.001
BMI (kg/m^2^)	31.8 ± 4.8	28.6 ± 4.0	<0.001
Plasma glucose, mmol/l			
fasting	6.6 ± 0.9	5.8 ± 0.5	<0.001
2-hour	9.9 ± 2.1	7.5 ± 1.5	<0.001
Serum insulin, pmol/l			
fasting	104.2 (76.4; 152.8)	69.5 (55.6; 90.3)	<0.001
2-hour	535 (363; 839)	347 (229; 514)	<0.001
Matsuda ISI	2.50 (1.69; 3.00) (N = 43)	4.26 (33.0; 5.72) (N = 40)	<0.001
DI_30_	71 (58.8; 86.5) (N = 43)	131 (105; 156) (N = 40)	<0.001

Data are mean ± SD or median (IQR). Matsuda ISI: Matsuda insulin sensitivity index DI_30_: disposition index

^*^P for the difference between groups using one-way ANOVA for continuous variables or χ^2^ test for categorical variable.

**Table 2:** Top ranking metabolites associated with T2D in lifestyle and groups* and their interaction with study group**.

	Study group	OR	Lower 95% CI	Higher 95% CI	P*	P**
Indolepropionic acid						0.19
	Lifestyle	0.46	0.28	0.76	0.002	
	Control	0.62	0.40	0.95	0.029	
PC(18:1/22:6)						0.02
	Lifestyle	0.34	0.19	0.59	2 × 10^−4^	
	Control	0.69	0.45	1.05	0.086	
LPC(19:0)						0.01
	Lifestyle	0.25	0.13	0.47	2 × 10^−5^	
	Control	0.65	0.42	1.01	0.058	
LPC(17:0)						0.19
	Lifestyle	0.33	0.19	0.58	1 × 10^−4^	
	Control	0.50	0.32	0.80	0.004	
LPC(20:1)						0.05
	Lifestyle	0.32	0.18	0.57	1 × 10^−4^	
	Control	0.66	0.43	1.02	0.059	
PC(22:6/18:2)						0.59
	Lifestyle	0.46	0.28	0.75	0.0021	
	Control	0.50	0.32	0.80	0.0037	
LPC(15:1)						0.09
	Lifestyle	0.41	0.24	0.69	7 × 10^−4^	
	Control	0.67	0.44	1.03	0.066	
PC(20:4/17:0)						0.38
	Lifestyle	0.41	0.24	0.70	0.001	
	Control	0.61	0.39	0.95	0.029	
PC(22:6/17:0)						0.95
	Lifestyle	0.57	0.36	0.91	0.019	
	Control	0.51	0.32	0.82	0.005	
PC(15:1/18:2)						0.87
	Lifestyle	0.58	0.37	0.92	0.020	
	Control	0.56	0.36	0.88	0.012	
LPE(16:0)						0.95
	Lifestyle	0.60	0.38	0.94	0.027	
	Control	0.56	0.36	0.87	0.011	
PC(18:2/15:0)						0.62
	Lifestyle	0.62	0.39	0.97	0.035	
	Control	0.53	0.34	0.84	0.006	
Tyrosine						0.01
	Lifestyle	1.38	0.89	2.12	0.15	
	Control	3.48	1.95	6.22	3 × 10^−5^	
Proline						0.001
	Lifestyle	0.99	0.65	1.50	0.96	
	Control	3.24	1.84	5.69	4 × 10^−5^	
Isoleucine						0.19
	Lifestyle	1.31	0.85	2.01	0.22	
	Control	2.20	1.36	3.55	0.0013	
Alanine						0.59
	Lifestyle	1.80	1.02	3.17	0.04	
	Control	2.45	1.33	4.54	0.004	
L-Phenylalanine						0.06
	Lifestyle	1.79	1.13	2.85	0.01	
	Control	3.26	1.85	5.74	4 × 10^−5^	

^*^Refers to the association of the respective metabolite with T2D in the unadjusted logistic regression in each of the study group (Lifestyle; control).

^**^Refers to the interaction of study group (lifestyle or control) vs. metabolite in the logistic regression testing the association of the respective metabolite with T2D adjusted for the study group.

**Table 3:** The effect of top ranking metabolites significantly associated with T2D on insulin sensitivity and insulin secretion during follow-up.

Metabolite	Traits (dependent variable)	Non-T2D cases (N = 104)				T2D cases (N = 96)			
		β	Lower 95% CI	Higher 95% CI	P^†^	β	Lower 95% CI	Higher 95% CI	P^†^
Indolepropionic acid	Matsuda ISI	0.13	−0.17	0.43	0.39	0.03	−0.22	0.27	0.83
	DI_30_	0.25	0.06	0.44	0.01	−0.04	−0.30	0.21	0.73
LPC(17:0)	Matsuda ISI	0.21	0.01	0.41	0.04	0.23	−0.02	0.47	0.07
	DI_30_	0.05	−0.15	0.25	0.60	0.15	−0.11	0.40	0.26
LPC(19:0)	Matsuda ISI	0.13	−0.07	0.33	0.20	0.12	−0.13	0.36	0.35
	DI_30_	−0.04	−0.24	0.16	0.69	0.07	−0.18	0.33	0.56
LPC(20:1)	Matsuda ISI	0.23	0.03	0.43	0.02	−0.01	−0.28	0.27	0.97
	DI_30_	−0.01	−0.21	0.19	0.91	0.15	−0.13	0.43	0.28
PC(22:6/18:2)	Matsuda ISI	0.21	0.01	0.40	0.04	−0.12	−0.36	0.12	0.31
	DI_30_	0.02	−0.18	0.22	0.82	0.05	−0.20	0.30	0.69
PC(18:1/22:6)	Matsuda ISI	0.15	−0.05	0.35	0.14	−0.05	−0.29	0.20	0.71
	DI_30_	−0.17	−0.36	0.03	0.10	0.19	−0.06	0.44	0.13
LPC(15:1)	Matsuda ISI	0.26	0.07	0.46	0.01	0.08	−0.17	0.32	0.54
	DI_30_	0.09	−0.11	0.30	0.37	0.05	−0.24	−0.34	0.73
PC(20:4/17:0)	Matsuda ISI	−0.04	−0.24	0.16	0.68	−0.02	−0.27	0.22	0.85
	DI_30_	0.03	−0.17	0.23	0.75	0.01	−0.24	0.27	0.92
PC(22:6/17:0)	Matsuda ISI	−0.07	−0.27	0.13	0.48	0.01	−0.23	0.26	0.91
	DI_30_	−0.16	−0.35	0.04	0.12	0.17	−0.09	0.42	0.19
PC(15:1/18:2)	Matsuda ISI	0.32	0.13	0.51	0.001	0.25	−0.01	0.51	0.06
	DI_30_	0.17	−0.03	0.36	0.10	0.00	−0.27	0.27	0.99
LPE(16:0)	Matsuda ISI	0.06	−0.14	0.26	0.57	−0.11	−0.36	0.15	0.40
	DI_30_	0.03	−0.17	0.23	0.79	−0.07	−0.33	0.20	0.61
PC(18:2/15:0)	Matsuda ISI	0.09	−0.11	0.30	0.37	0.14	−0.12	0.40	0.29
	DI_30_	0.15	−0.05	0.36	0.14	0.19	−0.08	0.45	0.16
L-Phenylalanine	Matsuda ISI	−0.23	−0.42	−0.04	0.02	−0.26	−0.49	−0.02	0.04
	DI_30_	−0.07	−0.27	0.13	0.48	0.04	−0.22	0.29	0.78
Tyrosine	Matsuda ISI	−0.36	−0.55	−0.17	2.7 × 10^−4^	−0.40	−0.63	−0.16	0.001
	DI_30_	−0.12	−0.32	0.08	0.23	0.06	−0.20	0.32	0.66
Alanine	Matsuda ISI	−0.08	−0.27	0.12	0.45	−0.03	−0.31	0.25	0.82
	DI_30_	0.17	−0.02	0.37	0.08	0.13	−0.15	0.42	0.35
Proline	Matsuda ISI	−0.18	−0.38	0.02	0.08	−0.08	−0.32	0.17	0.53
	DI_30_	−0.14	−0.34	0.06	0.18	0.09	−0.15	0.34	0.45
Isoleucine	Matsuda ISI	−0.27	−0.46	−0.08	0.006	−0.28	−0.53	−0.03	0.03
	DI_30_	−0.01	−0.21	0.19	0.90	0.02	−0.24	0.29	0.87

*Non-T2D cases mean of 7 years (up to 14 years). T2D cases up to 5 years.

^†^For ANCOVA models testing the effect of each metabolite on either one of the traits (dependent variable), adjusted for study group (fixed factor). Averaged measurements are calculated as: mean of years 2,3,4 and 5 in T2D cases and mean of years 2, 3, 4, 5 and 7 in non-T2D cases. ISI: insulin sensitivity index DI_30_: disposition index.
