# Supplementary material for: Indolepropionic acid and novel lipid metabolites are associated with a lower risk of type 2 diabetes in the Finnish Diabetes Prevention Study
Source: Sci Rep. 2017 Apr 11;7:46337. doi: 10.1038/srep46337 (PMC5387722; doi:10.1038/srep46337)
Supplement: Supplementary Information [file srep46337-s1.pdf]

Supplementary Material

Indolepropionic acid and novel lipid metabolites are associated with a lower risk of type 2 diabetes in the Finnish Diabetes Prevention Study

Vanessa D de Mello, Jussi Paananen, Jaana Lindström, Maria A Lankinen, Lin Shi, Johanna Kuusisto, Jussi Pihlajamäki, Seppo Auriola, Marko Lehtonen, Olov Rolandsson, Ingvar A Bergdahl, Elise Nordin, Pirjo Ilanne-Parikka, Sirkka Keinänen-Kiukaanniemi, Rikard Landberg, Johan G Eriksson, Jaakko Tuomilehto, Kati Hanhineva, Matti Uusitupa

Supplementary Methods

Diabetes Prevention Study (DPS) original design

The main inclusion criteria in the DPS were as follows: BMI>25 kg/m2, age 40-64 years, and impaired glucose tolerance (IGT) based on the mean values of two 75 g of glucose oral glucose tolerance tests (OGTT) based on the WHO 1985 criteria. People with previous diagnosis of type 2 diabetes (T2D), severe chronic disease or unstable clinical conditions related to glucose metabolism were excluded from the study. The study protocol was approved by the Ethics Committee of the National Public Health Institute of Helsinki, Finland, and all of the study individuals gave written informed consent.

The individuals in the intervention group received dietary tailored advice aiming at reducing weight and the intake of total and saturated fat and increasing the intake of dietary fiber, and instructions to increase their levels of physical activity. The main goals of the intervention group were weight reduction ≥ 5 %, moderate intensity physical activity ≥ 30 min/d, dietary fat < 30 % of total energy (E %), saturated fat < 10 E % and fiber ≥ 15 g/1000 kcal. The control group received general advice on the benefits of weight reduction, physical activity and healthy diet. The completeness of the food records was checked by the study nutritionist during each of the study visit. The mean daily nutrient intakes and food group/product intake were calculated with a dietary analysis program developed at the National Public Health Institute, Helsinki, Finland (3).

In the DPS the main end-point was diagnosis of diabetes defined by the WHO 1985 criteria (plasma fasting glucose ≥7.8 or 2-h glucose ≥11.1) to be confirmed by a repeated positive OGTT and verified by a physician. As explained in detail elsewhere (2, 3) after a median follow-up of four years as suggested by the independent endpoint committee the intervention phase of the study was discontinued. After the intervention (active study) period, the post-

intervention follow-up was carried out with annual examinations. As previously reported (3), of the original 522 participants, 366 individuals free of T2D participated in the post-intervention follow-up study at least once. They were further followed until diabetes diagnosis, dropout or the end of 2009 (median total follow-up of nine years and time span of 13 years from baseline). Of these, 62 new cases of diabetes out of 200 in the intervention group and 68 out of 166 in the control group were diagnosed. During this period, 36 participants withdrew and ten died without a verified diabetes diagnosis (3).

Reversed phase (RP) and hydrophilic interaction (HILIC) chromatography techniques in non-targeted LC-MS metabolite profiling analysis

In the RP technique, four microliters of the sample solution was injected onto the column (Zorbax Eclipse XDB-C18, 2.1 × 100mm, 1.8 µm, Agilent Technologies, Palo Alto, CA, USA) kept at 50 °C. The mobile phases, delivered at 0.4 mLl/min, consisted of water (eluent A, Milli-Q purified, Millipore, Milford, MA) and methanol (eluent B; Sigma-Aldrich, St. Louis, MO), both containing 0.1 % (v/v) of formic acid (Sigma-Aldrich, St. Louis, MO). The following gradient profile was employed: 0–10 min: 2% → 100% B,; 10–15 min: 100% B,; 15–15.1 min: 100% → 2% B; 15.1–18 min: 2% B.

In the HILIC technique, three µL of the sample solution was injected onto the column (Acquity UPLC BEH Amide column, 2.1 × 100mm, 1.7 µm) (Waters Corporation, Milford, MA) kept at 45 °C. The mobile phases, delivered at 0.6 mLl/min, consisted of 50% ACN (v/v)(eluent A) and 90% ACN (v/v)(eluent B) acetonitrile, respectively, both containing 20 mM ammonium formate, pH 3 (Sigma-Aldrich, St. Louis, MO). The following gradient profile was as employed: 0–2.5 min: 100% B, 2.5–10 min: 100% B → 0% B, 10–10.1 min: 0% B → 100% B; 10.1–14 min: 100% B.

The MS conditions after both chromatographic separations were as follows: Jetstream ESI source, operated in positive and negative ionization mode, drying gas temperature 325 °C and flow 10 L/min, sheath gas temperature 350 °C and flow 11 L/min, nebulizer pressure 45 psi, capillary voltage 3500 V, nozzle voltage 1000 V, fragmentor voltage 100 V, and skimmer 45 V. For data acquisition, 2 GHz extended dynamic range mode was used and the instrument was set to acquire over the m/z 50–1600. The data were collected in the centroid mode at the acquisition rate of 2.5 spectra/s (i.e. 400 ms/spectrum) with an abundance threshold 150. For the automatic data dependent MS/MS analyses performed on the QC samples four most abundant ions were selected for fragmentation from every precursor scan cycle. These ions were excluded after two product ion spectra and released again for fragmentation after a 0.25-min hold. The precursor scan time was based on ion intensity, ending at 20000 counts or after 300 ms. The product ion scan time was 300 ms. The collision energies were 10, 20 and 40 V in subsequent runs. The continuous mass axis calibration was performed by monitoring two reference ions from an infusion solution throughout the runs. The reference ions were m/z 121.050873 and m/z 922.009798 in positive mode and m/z 112.985587 and m/z 966.000725 in negative mode.

Calculations

Matsuda index of insulin sensitivity (ISI) was calculated as: 10,000 / square root of (fasting glucose x fasting insulin x [arithmetic mean of glucose x arithmetic mean insulin both during an OGTT at 0, 30, and 120 min]). Disposition index (DI_{30}) was calculated as the product of the ratio of total insulin area under the curve (AUC) and total glucose AUC during the 0-30min OGTT multiplied by the Matsuda ISI, as previously (4,5).

METSIM study

In the prospective population-based METSIM cohort the average follow-up time was 5.9 years and the diagnoses of T2D were based on an OGTT, HbA1c measurements and National Drug Reimbursement registry data. Indolepropionic acid data derived from the non-targeted metabolomics analyses were available from baseline and follow-up from 55 men who developed diabetes and 55 non-diabetic controls. At baseline, the means ± SD for BMI and fasting plasma glucose, respectively, were 59 ± 6 years, 29.5 ± 3.8 kg/m2 and 6.2 ± 0.5 mmol/l in cases and 59 ± 5 years, 26.0 ± 2.7 kg/m2 and 5.2 ± 0.2 mmol/l in controls.

Supplementary References

1. Eriksson J, Lindström J, Valle T, et al. (1999) Prevention of type II diabetes in subjects with impaired glucose tolerance: The diabetes prevention study (DPS) in Finland. Study design and 1-year interim report on the feasibility of the lifestyle intervention programme. Diabetologia 42:793-801, 1999

2. Tuomilehto J, Lindström J, Eriksson JG, et al. (2001) Prevention of type 2 diabetes mellitus by changes in lifestyle among subjects with impaired glucose tolerance. N Engl J Med 344:1343-1350

3. Lindström J, Peltonen M, Eriksson JG, et al. (2013) Improved lifestyle and decreased diabetes risk over 13 years: Long-term follow-up of the randomised finnish diabetes prevention study (DPS). Diabetologia 56:284-293

4. de Mello VD, Lindström J, Eriksson J, et al. (2012) Insulin secretion and its determinants in the progression of impaired glucose tolerance to type 2 diabetes in impaired glucose-tolerant individuals: the Finnish Diabetes Prevention Study. Diabetes Care 35:211-217

5. de Mello VD, Lindstrom J, Eriksson JG, et al. (2015) Markers of cholesterol metabolism as biomarkers in predicting diabetes in the Finnish Diabetes Prevention Study. Nutr Metab Cardiovasc Dis 25:635-642

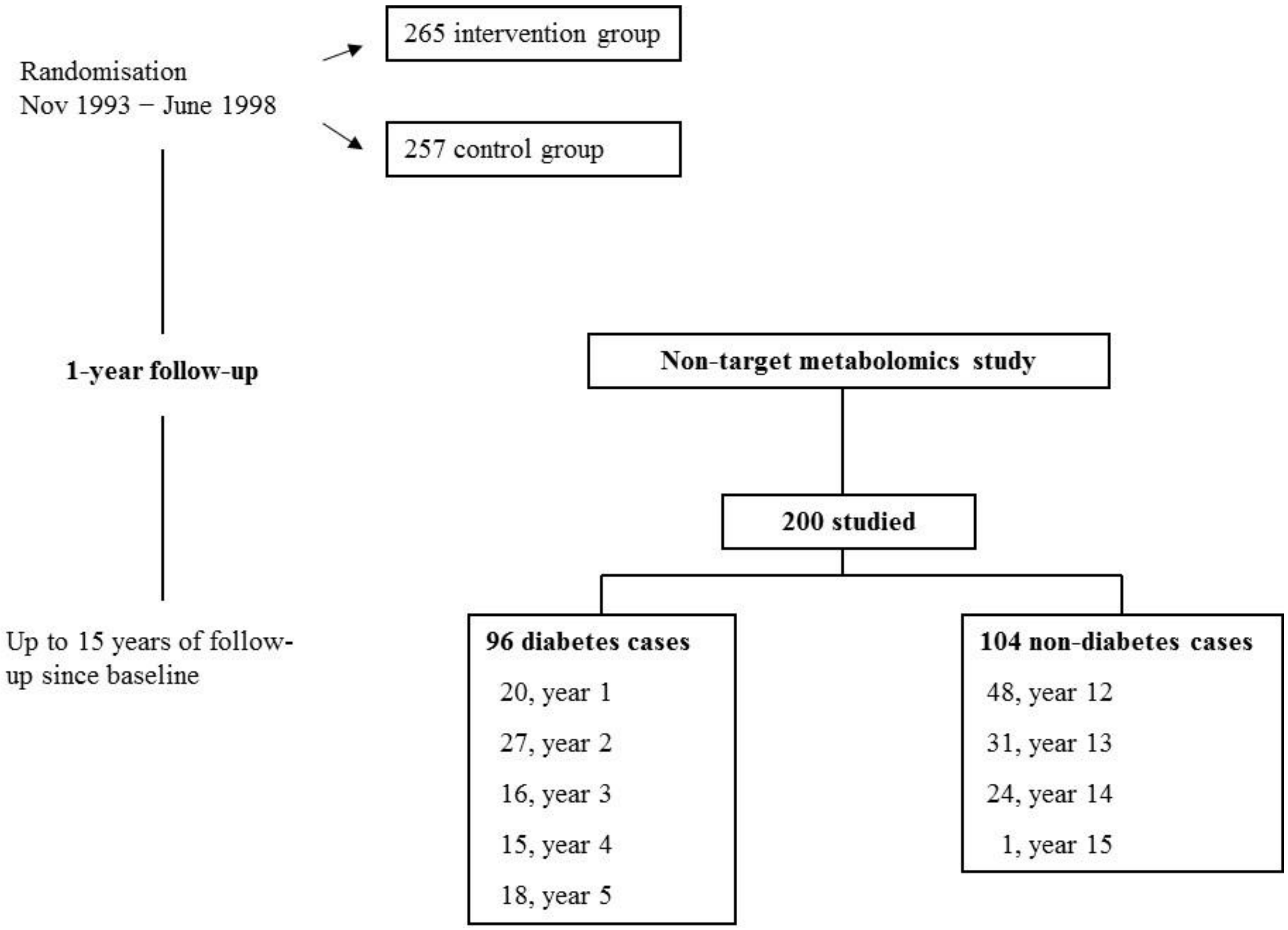

Supplementary Figure S1. Flowchart of metabolomics study in the Finnish Diabetes Prevention Study

Supplementary Table S1. Metabolites associated with T2D including statistically significantly unknown compounds and information on their compound class, analytical mode, retention time, molecular mass, and LC-MS/MS fragment ions used for structural assignment listed

Metabolite	Class	Mass	ms/ms	Mode	rt	OR	Lower 95%CI	Higher 95%CI	P*	FDR-P
Indolepropionic acid[S]	other	189.079	ESI(+) 190.085, 172.075, 130.064	RP+	5.66	0.55	0.40	0.76	$2.0x10^{-4}$	0.02
Betaine[S]	other	117.079	ESI(+) 118.086, 59.074, 58.066	hilic+	3.50	0.74	0.55	0.99	0.047	0.34
LPC(17:0)	lipid	509.348	ESI(+) 510.352, 184.073, 104.107; ESI(-) 554.346, 494.325, 269.248	RP+	10.34	0.42	0.29	0.59	$1.1x10^{-6}$	0.003
LPC(19:0)	lipid	537.377	ESI(-) 582.372, 522.359, 297.280	RP+	10.71	0.44	0.31	0.62	$4.1x10^{-6}$	0.005
LPC(20:1)	lipid	549.378	ESI(+) 550.386, 184.072, 104.107; ESI(-) 594.380, 534.356, 309.279	RP+	10.60	0.48	0.34	0.68	$2.3x10^{-5}$	0.008
PC(22:6/18:2)	lipid	829.560	ESI(+) 830.565, 184.072; ESI(-) 874.560, 814.538, 327.233, 303.233, 279.232	RP+	11.86	0.48	0.34	0.68	$3.1x10^{-5}$	0.009
PC(18:1/22:6)	lipid	831.575	ESI(+) 832.583, 184.073; ESI(-) 876.574, 816.554, 327.234, 281.249	RP+	12.21	0.49	0.36	0.69	$2.9x10^{-5}$	0.009
LPC(15:1)	lipid	479.338	ESI(+) 480.343, 184.073, 104.106; ESI(-) 524.336, 464.314, 375.230, 239.237, 168.042	RP+	10.32	0.50	0.36	0.70	$4.9x10^{-5}$	0.01
PC(20:4/17:0)	lipid	795.582	ESI(+) 796.583, 184.071; ESI(-) 840.578, 303.234, 269.246	RP+	12.42	0.52	0.38	0.73	$1.0x10^{-4}$	0.01

PC(22:6/17:0)	lipid	819.574	ESI(+) 820.585, 184.073; ESI(-) 864.573, 327.237, 269.247	RP+	12.40	0.54	0.39	0.75	$2.0x10^{-4}$	0.02
PC(15:1/18:2)	lipid	741.566	ESI(+) 742.570, 184.072; ESI(-) 786.565, 726.544, 279.230, 239.240	RP+	12.56	0.56	0.40	0.77	$4.0x10^{-4}$	0.04
LPE(16:0)	lipid	453.380	ESI(-) 452.278, 255.234, 196.038	RP+	9.58	0.55	0.40	0.76	$3.0x10^{-4}$	0.03
PC(18:2/15:0)	lipid	743.546	ESI(+) 744.551, 184.072; ESI(-) 788.542, 279.233, 241.217	RP+	12.02	0.57	0.42	0.79	$6.0x10^{-4}$	0.048
LPC(18:1)	lipid	521.348	ESI(+) 522.354, 184.072, 104.107; ESI(-) 566.345, 506.324, 281.249	RP+	10.25	0.59	0.44	0.81	$9.0x10^{-4}$	0.06
LPC(15:0)	lipid	481.316	ESI(+) 482.323, 464.313, 184.073, 125.000, 104.106; ESI(-) 526.314, 466.294, 241.21	RP+	9.88	0.58	0.42	0.81	0.001	0.06
PC(18:2/17:0)	lipid	771.576	ESI(+) 772.582, 184.074; ESI(-) 816.569, 756.555, 279.232, 269.248	RP+	12.60	0.59	0.43	0.81	0.001	0.06
PC(22:6/16:0)	lipid	805.562	ESI(+) 806.567, 184.072; ESI(-) 850.562, 790.541, 327.234, 283.241, 255.232	RP+	12.11	0.62	0.45	0.84	0.002	0.09
PC(20:3/18:0)	lipid	811.607	ESI(+) 812.613, 184.073; ESI(-) 856.608, 796.586, 305.248, 283.266	RP+	13.25	1.58	1.16	2.15	0.004	0.12
LPE(18:0)	lipid	481.352	ESI(+) 482.323, 464.306, 341.301; ESI(-) 480.309, 283.264, 196.039	RP+	10.40	0.67	0.49	0.90	0.009	0.17
LPC(18:2)	lipid	519.332	ESI(+) 520.339, 184.073, 104.108; ESI(-) 564.331, 504.309, 279.232	RP+	9.99	0.67	0.50	0.91	0.009	0.17

PC(14:0/18:1)	lipid	777.553	ESI(+) 732.550, 184.073; ESI(-) 776.543, 281.248, 253.217, 227.200	RP-	12.07	1.58	1.17	2.15	0.003	0.19
L-Phenylalanine[S]	AA	165.079	ESI(-) 164.071, 147.046, 126.209, 103.055, 91.053, 72.008	hilic+	3.62	2.24	1.59	3.15	$3.6x10^{-6}$	0.000 3
Alanine[S]	AA	43.042	ESI(+) 90.055, 44.050	hilic+	5.43	2.16	1.42	3.29	$3.0x10^{-4}$	0.02
Tyrosine[S]	AA	181.074	ESI(+) 182.079, 165.056, 147.046, 136.076, 123.045	hilic+	4.87	2.00	1.44	2.79	$3.7x10^{-5}$	0.002
Proline[S]	AA	115.064	ESI(+) 116.070, 70.065	hilic+	4.60	1.69	1.24	2.30	$8.0x10^{-4}$	0.03
Isoleucine[S]	AA	131.095	ESI(+) 132.103, 86.096, 69.070, 44.051	hilic+	3.97	1.63	1.20	2.21	0.002	0.049
Leucine[S]	AA	131.095	ESI(+) 132.103, 103.956, 86.096, 62.928, 44.049	hilic+	3.71	1.57	1.16	2.12	0.004	0.09
Phe-Phe	AA	312.147	ESI(+) 313.154, 166.086, 120.0811	hilic+	1.64	1.49	1.10	2.02	0.009	0.15
GCA[S]	BA	465.308	ESI(-) 464.303, 402.300; ESI(+) 448.300, 430.296, 412.285, 337.249, 319.237	RP-	8.47	1.81	1.30	2.53	$5.0x10^{-4}$	0.07
TCDC[S]	BA	499.296	ESI(-) 498.286; ESI(+) 464.283, 339.270	RP-	9.27	1.64	1.19	2.26	0.003	0.17
GCDC[S]	BA	449.315	ESI(+) 432.313, 414.302; ESI(-) 448.313, 386.304, 346.050, 74.023	RP-	8.96	1.60	1.17	2.18	0.003	0.17
GDC[S]	BA	449.314	ESI(+) 432.309, 414.299, 339.263, 278.168; ESI(-) 448.304	RP-	9.10	1.46	1.07	1.98	0.02	0.37
DC[S]	BA	392.292	ESI(-) 391.285, 345.276; ESI(+) 357.278, 339.267	RP-	9.60	1.43	1.06	1.94	0.02	0.39
CA[S]	BA	408.288	ESI(-) 407.281, 343.265, 289.219; ESI(+) 426.325, 373.277, 355.266	RP-	9.01	1.42	1.03	1.97	0.03	0.43

Unknown	lipid	529.329	Hilic+	1.15	0.45	0.32	0.64	$5.76x10^{-6}$	$4x10^{-4}$
Unknown	lipid	351.157	Hilic+	1.69	2.12	1.51	2.97	$1.54x10^{-5}$	0.001
Unknown	lipid	131.980	Hilic+	1.71	1.94	1.40	2.69	$6.02x10^{-5}$	0.004
Unknown	lipid	172.994	Hilic-	1.75	1.94	1.41	2.68	$5.17x10^{-5}$	0.001
Unknown	lipid	335.083	Hilic-	3.70	1.66	1.22	2.25	0.001	0.01
Unknown	lipid	249.968	Hilic-	4.43	1.61	1.18	2.18	0.002	0.02
Unknown	lipid	514.812	RP+	10.14	0.53	0.38	0.73	$1.00x10^{-4}$	0.02
Unknown	lipid	623.341	RP-	10.33	0.48	0.34	0.67	$1.39x10^{-5}$	0.01
Unknown	lipid	587.361	RP-	10.33	0.50	0.36	0.69	$3.03x10^{-5}$	0.01
Unknown	lipid	535.359	RP+	10.45	0.45	0.31	0.64	$1.71x10^{-5}$	0.01
Unknown	lipid	509.383	RP+	10.75	0.54	0.39	0.74	$2.00x10^{-4}$	0.02
Unknown	lipid	567.418	RP+	11.40	0.51	0.37	0.72	$9.35x10^{-5}$	0.02

* Logistic regression adjusted for study group (lifestyle or control) for the association of each metabolite with type 2 diabetes (cases and non-cases). FDR: correction for multiple comparisons was applied within each analytical mode including all the detected metabolite signals. AA: amino acid BA: bile acid Phe: phenylalanine rt: retention time GCA: Glycocholic acid TCDC: Taurochenodeoxycholic acid GCDC: Glycochenodeoxycholic acid GDC: Glycodeoxycholic DC: Deoxycholic acid CA: Cholic acid; upperscript S denotes compounds identified based on comparison with pure chemical standard.

Supplementary Table S2. Identified metabolites and their association with the development of type 2 diabetes in the Finnish Diabetes Prevention Study after excluding participants with diabetes at metabolomics sampling

Metabolite	Class	OR	Lower 95%CI	Higher 95%CI	P*	FDR-P*
Indolepropionic acid	other	0.55	0.39	0.78	$6.0x10^{-4}$	0.09
Betaine	other	0.72	0.52	0.98	0.039	0.44
LPC(17:0)	lipid	0.45	0.32	0.65	$1.5x10^{-5}$	0.03
LPC(19:0)	lipid	0.45	0.31	0.66	$2.5x10^{-5}$	0.03
LPC(20:1)	lipid	0.52	0.36	0.73	$2.0x10^{-4}$	0.06
PC(22:6/18:2)	lipid	0.52	0.37	0.74	$3.0x10^{-4}$	0.08
PC(18:1/22:6)	lipid	0.50	0.36	0.71	$1.0x10^{-4}$	0.06
LPC(15:1)	lipid	0.54	0.38	0.76	$4.0x10^{-4}$	0.08
PC(20:4/17:0)	lipid	0.53	0.37	0.75	$4.0x10^{-4}$	0.08
PC(22:6/17:0)	lipid	0.56	0.40	0.78	$7.0x10^{-4}$	0.09
PC(15:1/18:2)	lipid	0.55	0.39	0.78	$7.0x10^{-4}$	0.09
LPE(16:0)	lipid	0.55	0.39	0.77	$4.0x10^{-4}$	0.07
PC(18:2/15:0)	lipid	0.62	0.45	0.85	$4.1x10^{-3}$	0.20
LPC(18:1)	lipid	0.63	0.46	0.87	$5.4x10^{-3}$	0.20
LPC(15:0)	lipid	0.59	0.42	0.83	$2.3x10^{-3}$	0.18
PC(18:2/17:0)	lipid	0.62	0.45	0.86	$4.5x10^{-3}$	0.20
PC(22:6/16:0)	lipid	0.62	0.45	0.86	$3.8x10^{-3}$	0.20
PC(20:3/18:0)	lipid	1.45	1.06	2.00	0.022	0.31
LPE(18:0)	lipid	0.68	0.49	0.93	0.017	0.30
LPC(18:2)	lipid	0.71	0.52	0.97	0.031	0.32

PC(14:0/18:1)	lipid	1.56	1.13	2.16	$6.8x10^{-3}$	0.43
L-Phenylalanine	amino acid	1.92	1.37	2.70	$4.7x10^{-5}$	0.001
Tyrosine	amino acid	1.71	1.23	2.79	$1.5x10^{-3}$	0.07
Alanine	amino acid	2.16	1.40	3.33	$1.0x10^{-4}$	0.01
Proline	amino acid	1.58	1.15	2.18	$4.8x10^{-3}$	0.15
Isoleucine	amino acid	1.44	1.05	1.97	0.022	0.35
Phe-phe	amino acid	1.39	1.01	1.90	0.041	0.45
Leucine	amino acid	1.37	1.00	1.87	0.046	0.47
GCA	bile acid	1.78	1.26	2.52	$1.2x10^{-3}$	0.21
TCDC	bile acid	1.61	1.15	2.26	$5.5x10^{-3}$	0.41
GCDC	bile acid	1.59	1.20	2.20	$5.4x10^{-3}$	0.41
GDC	bile acid	1.47	1.07	2.04	0.019	0.59
DC	bile acid	1.53	1.10	2.12	$8.1x10^{-3}$	0.49
CA	bile acid	1.56	1.10	2.20	0.012	0.37

* Logistic regression adjusted for study group (lifestyle or control) for the association of each metabolite with type 2 diabetes (cases and non-cases). FDR: correction for multiple comparisons was applied within each analytical mode including all the detected metabolite signals. Phe: phenylalanine GCA: Glycocholic acid TCDC: Taurochenodeoxycholic acid GCDC: Glycochenodeoxycholic acid GDC: Glycodeoxycholic DC: Deoxycholic acid CA: Cholic acid

Supplementary Table S3. List of identified metabolites and their association with the development of type 2 diabetes in the Finnish Diabetes Prevention Study (N=200) in models further adjusted for BMI or sex

Metabolite	OR	Lower 95%CI	Higher 95%CI	P*	FDR-P*	OR	Lower 95%CI	Higher 95%CI	P†	FDR-P†
Indolepropionic acid	0.59	0.42	0.83	$2.5x10^{-3}$	0.21	0.55	0.40	0.76	$2.0x10^{-4}$	0.02
Betaine	0.84	0.62	1.15	0.28	0.88	0.69	0.50	0.95	0.02	0.23
LPC(17:0)	0.50	0.34	0.72	$3.0x10^{-4}$	0.13	0.42	0.29	0.59	$1.2x10^{-6}$	$3.2x10^{-3}$
LPC(19:0)	0.54	0.37	0.77	$9.0x10^{-4}$	0.14	0.44	0.31	0.62	$4.1x10^{-6}$	$3.8x10^{-3}$
LPC(20:1)	0.58	0.41	0.82	$2.4x10^{-3}$	0.21	0.48	0.34	0.67	$2.2x10^{-5}$	$7.4x10^{-3}$
PC(22:6/18:2)	0.56	0.39	0.79	$8.9x10^{-4}$	0.14	0.48	0.34	0.68	$2.9x10^{-5}$	$8x10^{-3}$
PC(18:1/22:6)	0.53	0.37	0.75	$3.7x10^{-4}$	0.13	0.49	0.36	0.69	$2.9x10^{-5}$	$8x10^{-3}$
LPC(15:1)	0.58	0.41	0.83	$2.3x10^{-3}$	0.21	0.50	0.36	0.70	$5.0x10^{-5}$	0.01
PC(20:4/17:0)	0.53	0.37	0.75	$3.5x10^{-4}$	0.13	0.51	0.36	0.72	$1.0x10^{-4}$	0.02
PC(22:6/17:0)	0.57	0.40	0.79	$9.3x10^{-4}$	0.14	0.54	0.39	0.75	$2.0x10^{-4}$	0.02
PC(15:1/18:2)	0.66	0.47	0.92	0.01	0.40	0.56	0.40	0.77	$4.0x10^{-4}$	0.04
LPE(16:0)	0.59	0.43	0.83	$2.1x10^{-3}$	0.21	0.54	0.39	0.75	$2.0x10^{-4}$	0.02

PC(18:2/15:0)	0.60	0.43	0.83	$2.2x10^{-3}$	0.21	0.55	0.40	0.77	$4.0x10^{-4}$	0.04
LPC(18:1)	0.72	0.51	1.00	0.05	0.57	0.59	0.43	0.80	$9.0x10^{-4}$	0.05
LPC(15:0)	0.64	0.46	0.90	$9.4x10^{-3}$	0.34	0.58	0.42	0.81	$1.0x10^{-3}$	0.05
PC(18:2/17:0)	0.65	0.47	0.90	$9.7x10^{-3}$	0.34	0.59	0.43	0.81	$1.1x10^{-3}$	0.05
PC(22:6/16:0)	0.63	0.46	0.87	$5.6x10^{-3}$	0.32	0.62	0.45	0.84	$2.1x10^{-3}$	0.08
PC(20:3/18:0)	1.41	1.01	1.96	0.04	0.57	1.64	1.19	2.25	$2.4x10^{-3}$	0.09
LPE(18:0)	0.78	0.56	1.07	0.12	0.61	0.66	0.49	0.90	$8.2x10^{-3}$	0.15
LPC(18:2)	0.84	0.60	1.16	0.29	0.81	0.65	0.47	0.89	$6.7x10^{-3}$	0.14
PC(14:0/18:1)	1.41	1.02	1.95	0.04	0.65	1.62	1.18	2.21	$2.5x10^{-3}$	0.16
L-Phenylalanine	1.97	1.39	2.81	$1.5x10^{-4}$	0.01	2.34	1.64	3.33	$2.6x10^{-6}$	$2.4x10^{-4}$
Tyrosine	1.75	1.24	2.46	$1.3x10^{-3}$	0.06	2.06	1.47	2.89	$2.9x10^{-5}$	$1.8x10^{-3}$
Alanine	2.18	1.42	3.34	$3.9x10^{-4}$	0.02	2.16	1.42	3.29	$3.0x10^{-4}$	0.02
Proline	1.67	1.21	2.31	$1.7x10^{-3}$	0.07	1.74	1.26	2.40	$7.0x10^{-4}$	0.03
Isoleucine	1.50	1.09	2.07	0.01	0.25	1.76	1.26	2.47	$9.0x10^{-4}$	0.03
Leucine	1.46	1.05	2.02	0.02	0.34	1.72	1.22	2.42	$1.9x10^{-3}$	0.05
Phe-phe	1.44	1.04	1.99	0.03	0.37	1.51	1.11	2.04	$8.2x10^{-3}$	0.14

GCA	1.75	1.23	2.48	$1.8x10^{-3}$	0.38	1.83	1.30	2.57	$5.0x10^{-4}$	0.06
TCDC	1.54	1.10	2.17	0.01	0.59	1.64	1.19	2.26	$2.8x10^{-3}$	0.16
GCDC	1.50	1.09	2.08	0.01	0.59	1.60	1.17	2.19	$3.0x10^{-3}$	0.17
GDC	1.44	1.04	1.99	0.03	0.61	1.46	1.07	1.98	0.02	0.35
DC	1.41	1.01	1.95	0.04	0.66	1.43	1.06	1.94	0.02	0.38
CA	1.45	1.03	2.04	0.03	0.64	1.42	1.03	1.97	0.03	0.43

[*] Logistic regression adjusted for study group (lifestyle or control) and body weight for the association of each metabolite with type 2 diabetes (cases and non-cases). [†] Logistic regression adjusted for study group (lifestyle or control) and sex for the association of each metabolite with type 2 diabetes (cases and non-cases). FDR: correction for multiple comparisons was applied within each analytical mode including all the detected metabolite signals. Phe: phenylalanine GCA: Glycocholic acid TCDC: Taurochenodeoxycholic acid GCDC: Glycochenodeoxycholic acid GDC: Glycodeoxycholic DC: Deoxycholic acid CA: Cholic acid.

Supplementary Table S4. List of identified metabolites and their association with the development of type 2 diabetes in the Finnish Diabetes Prevention Study (N=200) in models further adjusted for fasting or 2-h glucose during an oral glucose tolerance test at metabolomics sampling

Metabolite	OR	Lower 95%CI	Higher 95%CI	P*	FDR-P*	OR	Lower 95%CI	Higher 95%CI	P†	FDR-P†
Indolepropionic acid	0.58	0.40	0.83	$3.1x10^{-3}$	0.28	0.61	0.43	0.89	$9.2x10^{-3}$	0.36
Betaine	0.66	0.47	0.93	0.20	0.35	0.85	0.60	1.20	0.35	0.82
LPC(17:0)	0.51	0.35	0.75	$6.6x10^{-4}$	0.21	0.46	0.31	0.70	$2.2x10^{-4}$	0.18
LPC(19:0)	0.49	0.33	0.72	$2.5x10^{-4}$	0.21	0.42	0.28	0.64	$4.7x10^{-5}$	0.13
LPC(20:1)	0.53	0.37	0.78	$1.2x10^{-3}$	0.21	0.53	0.36	0.78	$1.5x10^{-3}$	0.36
PC(22:6/18:2)	0.50	0.33	0.73	$4.3x10^{-4}$	0.21	0.58	0.39	0.86	$6.3x10^{-3}$	0.36
PC(18:1/22:6)	0.56	0.38	0.81	$2.2x10^{-3}$	0.28	0.51	0.34	0.75	$7.4x10^{-4}$	0.34
LPC(15:1)	0.58	0.41	0.84	$4.0x10^{-3}$	0.29	0.55	0.37	0.80	$2.3x10^{-3}$	0.36
PC(20:4/17:0)	0.54	0.37	0.78	$1.1x10^{-3}$	0.21	0.54	0.37	0.79	$1.6x10^{-3}$	0.36
PC(22:6/17:0)	0.61	0.43	0.86	$5.3x10^{-3}$	0.31	0.53	0.37	0.78	$1.1x10^{-3}$	0.36
PC(15:1/18:2)	0.60	0.42	0.87	$6.9x10^{-3}$	0.33	0.63	0.44	0.92	0.02	0.36

LPE(16:0)	0.51	0.35	0.74	$4.3x10^{-4}$	0.21	0.62	0.42	0.90	0.01	0.36
PC(18:2/15:0)	0.76	0.54	1.07	0.11	0.48	0.61	0.42	0.88	$9.0x10^{-3}$	0.36
LPC(18:1)	0.64	0.45	0.91	0.01	0.36	0.68	0.47	0.98	0.04	0.37
LPC(15:0)	0.70	0.49	1.00	0.05	0.36	0.67	0.45	0.98	0.04	0.38
PC(18:2/17:0)	0.66	0.47	0.94	0.02	0.36	0.71	0.49	1.02	0.07	0.44
PC(22:6/16:0)	0.67	0.47	0.94	0.02	0.36	0.61	0.43	0.88	$8.6x10^{-3}$	0.36
PC(20:3/18:0)	1.51	1.06	2.15	0.02	0.36	1.31	0.91	1.87	0.14	0.55
LPE(18:0)	0.75	0.53	1.07	0.11	0.47	0.65	0.45	0.94	0.02	0.36
LPC(18:2)	0.76	0.54	1.07	0.11	0.48	0.61	0.42	0.88	$8.7x10^{-3}$	0.36
PC(14:0/18:1)	1.59	1.12	2.27	0.01	0.70	1.41	0.99	2.00	0.06	0.91
L-Phenylalanine	2.08	1.41	3.06	$2.0x10^{-4}$	0.15	1.55	1.05	2.28	0.03	0.36
Tyrosine	1.64	1.16	2.34	$6.0x10^{-3}$	0.35	1.38	0.96	1.99	0.08	0.52
Alanine	2.49	1.57	3.95	$1.0x10^{-4}$	0.15	1.90	1.17	3.07	0.01	0.24
Proline	1.50	1.08	2.09	0.02	0.35	1.55	1.10	2.18	0.01	0.26
Isoleucine	1.48	1.04	2.09	0.03	0.35	1.30	0.91	1.86	0.14	0.60
Leucine	1.44	1.01	2.05	0.04	0.35	1.22	0.85	1.75	0.27	0.76

Phe-phe	1.40	0.99	1.99	0.06	0.36	1.24	0.87	1.78	0.24	0.73
GCA	1.73	1.18	2.52	$4.7x10^{-3}$	0.61	1.66	1.12	2.46	0.01	0.81
TCDC	1.50	1.04	2.16	0.03	0.81	1.55	1.05	2.29	0.03	0.83
GCDC	1.38	0.98	1.94	0.07	0.92	1.46	1.02	2.09	0.04	0.90
GDC	1.32	0.94	1.87	0.11	0.94	1.34	0.93	1.94	0.12	0.96
DC	1.21	0.86	1.70	0.28	1.00	1.62	1.09	2.41	0.02	0.81
CA	1.24	0.85	1.80	0.27	1.00	1.45	1.01	2.10	0.046	0.91

[*] Logistic regression adjusted for study group (lifestyle or control) and fasting glucose for the association of each metabolite with type 2 diabetes (cases and non-cases). [†] Logistic regression adjusted for study group (lifestyle or control) and 2-h glucose for the association of each metabolite with type 2 diabetes (cases and non-cases). FDR: correction for multiple comparisons was applied within each analytical mode including all the detected metabolite signals. Phe: phenylalanine GCA: Glycocholic acid TCDC: Taurochenodeoxycholic acid GCDC: Glycochenodeoxycholic acid GDC: Glycodeoxycholic DC: Deoxycholic acid CA: Cholic acid.

Supplementary Table S5. List of identified metabolites and their association with the development of type 2 diabetes in the Finnish Diabetes Prevention Study (N=200) in models further adjusted for fasting or 2-h insulin during an oral glucose tolerance test at metabolomics sampling

Metabolite	OR	Lower 95%CI	Higher 95%CI	P*	FDR-P*	OR	Lower 95%CI	Higher 95%CI	P†	FDR-P†
Indolepropionic acid	0.40	0.80	0.57	$1.0x10^{-3}$	0.23	0.39	0.79	0.56	$1.1x10^{-3}$	0.20
Betaine	0.58	1.06	0.78	0.12	0.78	0.62	1.16	0.85	0.30	0.79
LPC(17:0)	0.31	0.66	0.45	$3.1x10^{-5}$	0.08	0.30	0.62	0.43	$8.9x10^{-6}$	0.02
LPC(19:0)	0.33	0.68	0.48	$5.9x10^{-5}$	0.08	0.32	0.66	0.46	$2.6x10^{-5}$	0.04
LPC(20:1)	0.38	0.76	0.53	$4.0x10^{-4}$	0.22	0.35	0.72	0.50	$2.0x10^{-4}$	0.09
PC(22:6/18:2)	0.37	0.76	0.53	$4.8x10^{-4}$	0.22	0.34	0.72	0.50	$2.0x10^{-4}$	0.09
PC(18:1/22:6)	0.38	0.76	0.54	$3.8x10^{-4}$	0.22	0.38	0.77	0.54	$6.0x10^{-4}$	0.17
LPC(15:1)	0.40	0.81	0.57	$1.6x10^{-3}$	0.28	0.42	0.83	0.59	$2.2x10^{-3}$	0.23
PC(20:4/17:0)	0.40	0.80	0.56	$1.1x10^{-3}$	0.23	0.40	0.79	0.56	$1.1x10^{-3}$	0.20
PC(22:6/17:0)	0.41	0.80	0.57	$1.0x10^{-3}$	0.23	0.38	0.77	0.54	$6.0x10^{-4}$	0.17
PC(15:1/18:2)	0.48	0.95	0.68	0.03	0.53	0.48	0.96	0.68	0.03	0.48

LPE(16:0)	0.42	0.81	0.58	$1.4x10^{-3}$	0.26	0.41	0.82	0.58	0.002	0.23
PC(18:2/15:0)	0.46	0.87	0.63	$5.6x10^{-3}$	0.35	0.45	0.87	0.62	$5.3x10^{-3}$	0.28
LPC(18:1)	0.47	0.90	0.65	$9.5x10^{-3}$	0.40	0.47	0.90	0.65	$8.9x10^{-3}$	0.34
LPC(15:0)	0.43	0.85	0.60	$3.9x10^{-3}$	0.29	0.41	0.83	0.59	$2.6x10^{-3}$	0.23
PC(18:2/17:0)	0.45	0.86	0.62	$3.7x10^{-3}$	0.29	0.44	0.87	0.62	$5.6x10^{-3}$	0.28
PC(22:6/16:0)	0.48	0.90	0.66	$9.2x10^{-3}$	0.40	0.48	0.91	0.66	0.01	0.39
PC(20:3/18:0)	0.99	1.92	1.37	0.06	0.57	0.92	1.78	1.28	0.14	0.60
LPE(18:0)	0.53	0.99	0.72	0.045	0.57	0.53	1.02	0.74	0.06	0.52
LPC(18:2)	0.54	1.01	0.74	0.06	0.57	0.54	1.01	0.74	0.06	0.52
PC(14:0/18:1)	1.08	2.06	1.49	0.02	0.79	1.07	2.07	1.49	0.02	0.69
L-Phenylalanine	1.85	1.29	2.67	$9.0x10^{-4}$	0.05	1.99	1.38	2.86	$2.0x10^{-4}$	0.01
Tyrosine	1.64	1.15	2.35	$7.0x10^{-3}$	0.29	1.73	1.21	2.48	$2.8x10^{-3}$	0.12
Alanine	1.92	1.26	2.93	$2.0x10^{-3}$	0.11	1.92	1.24	2.97	$3.3x10^{-3}$	0.14
Proline	1.43	1.04	1.97	0.03	0.53	1.55	1.13	2.12	$6.5x10^{-3}$	0.20
Isoleucine	1.31	0.94	1.83	0.11	0.76	1.37	0.98	1.90	0.06	0.57
Leucine	1.27	0.91	1.76	0.17	0.85	1.36	0.98	1.90	0.06	0.57

Phe-phe	1.31	0.95	1.81	0.10	0.73	1.32	0.96	1.83	0.09	0.63
GCA	1.60	1.13	2.26	$8.0x10^{-3}$	0.74	1.70	1.20	2.41	$3.0x10^{-3}$	0.37
TCDC	1.47	1.05	2.05	0.03	0.82	1.47	1.04	2.07	0.03	0.77
GCDC	1.44	1.05	1.97	0.03	0.83	1.52	1.10	2.10	0.01	0.60
GDC	1.35	0.98	1.85	0.06	0.92	1.41	1.02	1.95	0.04	0.78
DC	1.27	0.92	1.75	0.14	0.92	1.37	0.99	1.90	0.06	0.88
CA	1.33	0.95	1.86	0.10	0.92	1.41	1.00	1.99	0.05	0.85

[*] Logistic regression adjusted for study group (lifestyle or control) and fasting insulin for the association of each metabolite with type 2 diabetes (cases and non-cases). [†] Logistic regression adjusted for study group (lifestyle or control) and 2-h insulin for the association of each metabolite with type 2 diabetes (cases and non-cases). FDR: correction for multiple comparisons was applied within each analytical mode including all the detected metabolite signals. Phe: phenylalanine. GCA: Glycocholic acid TCDC: Taurochenodeoxycholic acid GCDC: Glycochenodeoxycholic acid GDC: Glycodeoxycholic DC: Deoxycholic acid CA: Cholic acid.

Supplementary Table S6. Associations of lipid metabolites related to lower chance of developing T2D with circulating high sensitive C-reactive protein (hsCRP) at metabolomics sampling adjusted for confounding factors

Metabolite		Model 1	Model 2	Model 3	Model 4
LPC(17:0)					
	β	-0.41	-0.38	-0.35	-0.41
	p	$3.1x10^{-7}$	$2.9x10^{-6}$	$3.4x10^{-5}$	$2.2x10^{-7}$
LPC(15:1)					
	β	-0.36	-0.33	-0.30	-0.37
	p	$6.3x10^{-6}$	$3.1x10^{-5}$	$2.0x10^{-4}$	$7.9x10^{-6}$
LPC(20:1)					
	β	-0.43	-0.39	-0.37	-0.43
	p	$7.5x10^{-8}$	$8.9x10^{-7}$	$1.1x10^{-5}$	$9.7x10^{-8}$
PC(22:6/18:2)					
	β	-0.29	-0.25	-0.24	-0.29
	p	$4.8x10^{-4}$	0.003	0.004	$4.5x10^{-4}$
PC(15:1/18:2)					
	β	-0.20	-0.15	-0.13	-0.20
	p	0.02	0.07	0.13	0.02

* Linear regression models testing the association of each lipid metabolite with hsCRP (dependent variable). adjusted for either one of the confounding factors: fasting glucose (Model 1). 2-h glucose (Model 2). BMI (Model 3) or study group (Model 4).

Supplementary Table S7. Correlations of identified top ranking metabolites with dietary intake[*]

		CHO	Fiber[†]	Fat	SAFA	MUFA	PUFA	Protein
Indolepropionic acid	r	0.28	0.23	-0.15	-0.10	-0.20	-0.10	-0.05
	P	$9.1x10^{-5}$	0.001	0.034	0.18	0.004	0.14	0.51
LPC(17:0)	r	0.09	0.07	0.01	-0.08	0.04	0.08	-0.03
	P	0.23	0.31	0.92	0.24	0.61	0.26	0.63
LPC(19:0)	r	0.11	0.12	-0.02	-0.24	0.06	0.19	0.01
	P	0.11	0.08	0.74	$8.0x10^{-4}$	0.41	0.01	0.93
LPC(20:1)	r	-0.04	0.05	0.05	-0.19	0.14	0.21	0.04
	P	0.54	0.49	0.46	0.009	0.05	0.004	0.61
PC(22:6/18:2)	r	-0.02	0.05	-0.02	-0.08	0.00	0.05	0.09
	P	0.79	0.46	0.80	0.26	0.99	0.50	0.22
PC(18:1/22:6)	r	0.14	0.17	-0.10	-0.22	-0.06	0.14	0.08
	P	0.06	0.02	0.15	0.002	0.43	0.05	0.28
LPC(15:1)	r	0.04	0.00	-0.06	-0.17	-0.02	0.04	-0.04
	P	0.59	0.95	0.37	0.02	0.79	0.58	0.59
PC(20:4/17:0)	r	0.04	0.08	-0.05	0.01	-0.09	-0.09	0.16
	P	0.62	0.29	0.45	0.94	0.20	0.23	0.03
PC(22:6/17:0)	r	0.18	0.23	-0.15	-0.16	-0.16	-0.04	0.14
	P	0.01	0.00	0.04	0.03	0.03	0.61	0.05
PC(15:1/18:2)	r	0.08	0.06	-0.08	-0.14	-0.04	0.01	0.02
	P	0.29	0.42	0.29	0.05	0.57	0.84	0.77

LPE(16:0)	r	-0.10	-0.09	0.14	0.03	0.16	0.10	0.01
	P	0.15	0.23	0.05	0.71	0.03	0.17	0.84
PC(18:2/15:0)	r	0.33	0.12	-0.14	-0.06	-0.17	-0.06	-0.09
	P	0.000	0.09	0.04	0.43	0.02	0.41	0.22
L_Phenylalanine	r	-0.10	-0.13	0.01	0.06	0.05	-0.11	0.10
	P	0.14	0.08	0.84	0.39	0.50	0.13	0.15
Tyrosine	r	-0.08	-0.03	-0.01	0.03	0.01	-0.09	0.13
	P	0.28	0.66	0.90	0.64	0.86	0.23	0.06
Alanine	r	-0.03	-0.13	0.04	0.10	0.04	-0.08	-0.05
	P	0.71	0.07	0.61	0.18	0.54	0.25	0.52
Proline	r	-0.02	0.03	0.02	-0.01	0.07	0.02	0.00
	P	0.80	0.65	0.79	0.85	0.31	0.82	0.97
Isoleucine	r	-0.13	-0.09	0.12	0.15	0.09	-0.12	0.06
	P	0.08	0.21	0.10	0.03	0.22	0.10	0.37

[*] Energy adjusted nutrient intake [†] Total fiber (g) per 1000 kcal energy intake CHO: carbohydrates SAFA: saturated fat MUFA: monounsaturated fat PUFA: polyunsaturated fat.

Supplementary Table S8. Pearson correlation (r) of indolepropionic acid with the most significant metabolites associated with type 2 diabetes in the Finnish Diabetes Prevention Study (n=200)

		Indolepropionic acid
LPC(17:0)	r	0.24
	P	0.001
LPC(19:0)	r	0.23
	P	0.001
LPC(20:1)	r	0.14
	P	0.06
PC(22:6/18:2)	r	0.03
	P	0.69
PC(18:1/22:6)	r	0.06
	P	0.39
LPC(15:1)	r	0.17
	P	0.01
PC(20:4/17:0)	r	0.03
	P	0.69
PC(22:6/17:0)	r	0.05
	P	0.46
PC(15:1/18:2)	r	0.09
	P	0.19
LPE(16:0)	r	0.08

	P	0.29
PC(18:2/15:0)	r	0.18
	P	0.01
L_Phenylalanine	r	-0.20
	P	0.005
Tyrosine	r	0.05
	P	0.46
Alanine	r	-0.08
	P	0.29
Proline	r	-0.04
	P	0.62
Isoleucine	r	-0.10
	P	0.17

Supplementary Table S9. Pearson correlation (r) between bile acids and the most significant metabolites associated with type 2 diabetes in the Finnish Diabetes Prevention Study (n=200)

		GCA	TCDC	GCDC	GDC	DC	CA
Indolepropionic acid	r	-0.07	-0.03	0.02	-0.05	-0.02	0.01
	P	0.36	0.65	0.75	0.46	0.74	0.86
LPC(17:0)	r	-0.16	-0.19	-0.16	-0.15	-0.02	0.10
	P	0.029	0.007	0.029	0.032	0.75	0.18
LPC(19:0)	r	-0.07	-0.12	-0.17	-0.06	-0.03	0.15
	P	0.35	0.10	0.02	0.44	0.64	0.04
LPC(20:1)	r	-0.02	-0.04	-0.10	-0.01	0.00	0.20
	P	0.76	0.55	0.15	0.90	0.96	0.004
PC(22:6/18)	r	-0.02	-0.15	-0.06	-0.02	0.00	0.08
	P	0.73	0.03	0.41	0.75	1.00	0.28
PC(18:1/22:6)	r	-0.10	-0.12	-0.05	-0.07	-0.06	0.04
	P	0.18	0.09	0.46	0.31	0.38	0.58
LPC(15:1)	r	-0.14	-0.19	-0.18	-0.11	-0.08	0.06
	P	0.06	0.01	0.01	0.12	0.27	0.40
PC(20:4/17:0)	r	-0.12	-0.12	0.03	-0.11	0.02	-0.01
	P	0.10	0.08	0.69	0.12	0.75	0.86
PC(22:6/17:0)	r	-0.14	-0.08	-0.02	-0.15	-0.06	-0.04
	P	0.05	0.27	0.73	0.03	0.39	0.56
PC(15:1/18:2)	r	-0.11	-0.16	-0.04	-0.09	-0.11	-0.10
	P	0.14	0.03	0.57	0.21	0.12	0.17

LPE(16:0)	r	-0.05	-0.15	-0.13	0.02	-0.17	0.08
	P	0.50	0.03	0.07	0.82	0.02	0.28
PC(18:2/15:0)	r	-0.15	-0.26	-0.03	-0.16	0.02	-0.09
	P	0.03	$2x10^{-4}$	0.69	0.03	0.78	0.23
L-Phenylalanine	r	0.20	0.12	0.16	0.13	0.09	0.05
	P	0.01	0.10	0.03	0.07	0.18	0.49
Tyrosine	r	0.22	0.15	0.25	0.19	0.13	0.12
	P	0.002	0.03	$4.4x10^{-4}$	0.01	0.07	0.10
Alanine	r	0.23	0.10	0.18	0.10	0.04	0.10
	P	0.001	0.15	0.01	0.16	0.53	0.17
Proline	r	0.26	0.09	0.27	0.20	0.15	0.21
	P	$1.8x10^{-4}$	0.19	$9.2x10^{-5}$	0.004	0.04	0.002
Isoleucine	r	0.20	0.08	0.21	0.13	0.07	0.10
	P	0.01	0.27	0.003	0.08	0.36	0.16

IPA: Indolepropionicacid GCA: Glycocholic acid TCDC: Taurochenodeoxycholic acid GCDC: Glycochenodeoxycholic acid GDC: Glycodeoxycholic DC: Deoxycholic acid CA: Cholic acid.
